# Could Gut Microbiota Composition Be a Useful Indicator of a Long-Term Dietary Pattern?

**DOI:** 10.3390/nu15092196

**Published:** 2023-05-05

**Authors:** Karin Šik Novak, Nives Bogataj Jontez, Ana Petelin, Matjaž Hladnik, Alenka Baruca Arbeiter, Dunja Bandelj, Jure Pražnikar, Saša Kenig, Nina Mohorko, Zala Jenko Pražnikar

**Affiliations:** 1 Faculty of Health Sciences, University of Primorska, Polje 42, 6310 Izola, Slovenia; 2 Faculty of Mathematics, Natural Sciences and Information Technologies, University of Primorska, Glagoljaška 8, 6000 Koper, Slovenia

**Keywords:** gut microbiota, dietary pattern, omnivorous, vegan, vegetarian, low-carbohydrate, high-fat

## Abstract

Despite the known effects of diet on gut microbiota composition, not many studies have evaluated the relationship between distinct dietary patterns and gut microbiota. The aim of our study was to determine whether gut microbiota composition could be a useful indicator of a long-term dietary pattern. We collected data from 89 subjects adhering to omnivorous, vegetarian, vegan, and low-carbohydrate, high-fat diet that were equally distributed between groups and homogenous by age, gender, and BMI. Gut microbiota composition was analyzed with a metabarcoding approach using V4 hypervariable region of the 16S rRNA gene. K-means clustering of gut microbiota at the genus level was performed and the nearest neighbor classifier was applied to predict microbiota clustering classes. Our results suggest that gut microbiota composition at the genus level is not a useful indicator of a subject’s dietary pattern, with the exception of a vegan diet that is represented by a high abundance of *Prevotella 9*. Based on our model, a combination of 26 variables (anthropometric measurements, serum biomarkers, lifestyle factors, gastrointestinal symptoms, psychological factors, specific nutrients intake) is more important to predict an individual’s microbiota composition cluster, with 91% accuracy, than the dietary intake alone. Our findings could serve to develop strategies to educate individuals about changes of some modifiable lifestyle factors, aiming to classify them into clusters with favorable health markers, independent of their dietary pattern.

## 1. Introduction

The gut microbiota is the largest microbial community in humans and its importance for human health is hard to estimate. Observational studies have observed its influences on human metabolic health, and different comparative surveys have demonstrated associations between metabolic disorders such as obesity, cardiovascular disease, and type 2 diabetes and the underrepresentation of certain commensal microbial taxa as well as the increased prevalence of potential pathobionts [1]. Gut microbiota mainly include prokaryotic species (bacteria) that can be taxonomically classified into kingdoms, phyla, classes, orders, families, genera, and species [2]. The phyla Bacteroidota and Firmicutes represent 90% of total gut microbiota, but other phyla, such as Actinobacteria, Proteobacteria, Fusobacteria, and Verrucomicrobia, are also frequently present [3].

A growing body of evidence accumulated by studies of gut microbiota in world populations emphasizes that lifestyle, and especially diet, strongly impacts microbiota composition and, thus, human health. In addition to most common omnivorous (O) diet, dietary patterns such as vegan (V), vegetarian (VE), and low-carbohydrate, high-fat (LCHF) diet have become popular recently [4]. It has been shown that the gut microbiota of adults that consume more animal protein is dominated by *Bacteroides*, whereas *Prevotella* is associated with a plant-based diet [2]. A high intake of saturated fatty acids (SFA), sugar, and salt promote the growth of pathogenic bacteria, and, contrarily, the intake of dietary fiber and plant protein increases the abundance of beneficial bacteria that promote the production of short-chain fatty acids (SCFAs) [5].

Despite the known effects of diet on shaping gut microbiota composition, not many studies have systematically evaluated the associations between dietary patterns and gut microbiota. In studies comparing O, VE, and V, a lower relative abundance of *Bacteroides* in V and VE [6], and a greater diversity of microbiota in V, compared to O, have been observed [7]. Additionally, a higher relative abundance of bacteria from the phylum Actinobacteria, a lower abundance of bacteria from the phylum Proteobacteria, and a higher ratio between the genera *Prevotella* and *Bacteroides* in VE, compared to O, have been reported [8]. The phylum Bacteroidota was dominant among all three diet groups, and a statistically significant difference in the abundance of Bacteroidota was observed between V and O, and VE and O [7].

On the contrary, a systematic review of the literature found no associations between V or VE diets and microbiota composition compared to O. Some studies show conflicting results, which could be due to differences between individuals and different methods used [9]. An LCHF diet has been associated with a lower relative abundance of *Bifidobacteria* and a higher abundance of *Akkermansia* and *E. coli* [5], but no studies have compared it to other dietary patterns. It seems that, more than by dietary pattern, gut microbiota could be shaped by the intake of specific nutrients [7]. Moreover, due to the immense variability in microbial composition at the species level, it is still not known what may constitute the elusive “golden standard” of a healthy gut microbiota [10].

The aim of the present study was to determine whether a long-term dietary pattern can impact the composition of gut microbiota at the genus level. In particular, we were interested in whether adherence to a particular dietary pattern alters the microbiota to such an extent that it is possible to determine which pattern a person adheres to based on their gut microbiota composition. For that purpose, dietary data were collected from a cohort of subjects adhering to O, VE, V, and LCHF diet that were equally distributed between groups and were homogenous by age, gender, and BMI. K-means clustering of the microbiota dataset at the genus level was performed, and the nearest neighbor classifier based on approximately two hundred variables was applied to predict the clustering classes of gut microbiota.

## 2. Materials and Methods

### 2.1. Study Design

**Figure 1:**
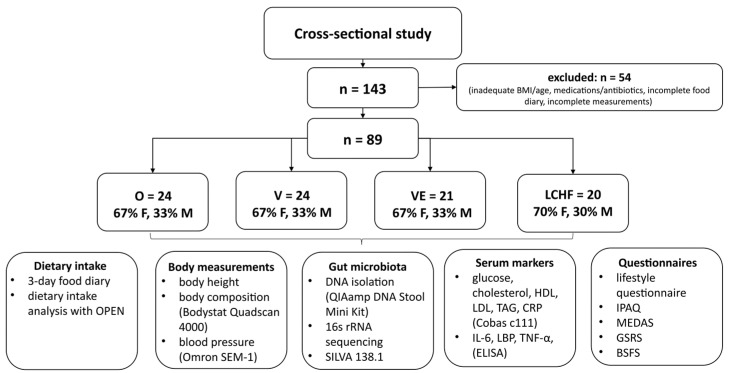
Study design. O—omnivorous; V—vegan; VE—vegetarian; LCHF—low-carbohydrate, high-fat; F—females; M—males; OPEN—Open Platform for Clinical Nutrition; HDL—high-density lipoprotein; LDL—low-density lipoprotein; TAG—triacylglycerol; CRP—C-reactive protein; IL-6—interleukin-6; LBP—lipopolysaccharide binding protein; TNF-α—tumor necrosis factor-α; IPAQ—International Physical Activity Questionnaire; MEDAS—Mediterranean Diet Adherence Score; GSRS—Gastrointestinal Symptoms Rating Scale; BSFS—Bristol Stool Form Scale.

To compare gut microbiota composition and other health-related markers in subjects with distinct dietary patterns, we performed a cross-sectional study named “The Link between Diets and Health Indicators (DIETE)” that lasted from February 2020 to October 2021. The study protocol was approved by the Slovenian National Medical Ethics Committee (No. 0120-557/2017/4 and 53/03/15) and was registered on ClinicalTrials.gov (Identifier: NCT04347213), accessed on 15 April 2020. The study design is presented in Figure 1.

### 2.2. Study Subjects

Subjects with four distinct dietary patterns (O, V, VE, LCHF) were recruited through a post in newspapers and on social media in different targeted groups. The subjects were asymptomatic, aged from 20 to 60 years, with a BMI of 18.5 to 30 kg/m^2^ and an unchanged eating pattern for at least 6 months prior to participation in the study. The exclusion criteria were (a) taking medications or antibiotics 3 months prior to participation, (b) being pregnant or lactating, and (c) a significant change in body mass 3 months prior to participation. The required sample size to compare four groups of subjects, that was calculated a priori using G*Power 3.1.9.7 (Heinrich-Heine-Universität Düsseldorf, Germany), assuming an α level of 5% and β level of 20% and a medium effect size (d = 0.4), was *n* = 76. Overall, a total of 89 subjects fulfilled the inclusion criteria to participate in the present study. Others (*n* = 54) were excluded from the study due to not meeting the inclusion criteria or due to incomplete measurements (Figure 1). The subjects were equally distributed between groups and were homogenous by age, gender, and BMI.

### 2.3. Anthropometric Measurements

Anthropometric measurements were performed in the morning after fasting and refraining from physical exercise for at least 12 h in standardized conditions. Systolic blood pressure, diastolic blood pressure, and heart rate were measured on the left upper arm, in a seated position, with an automatic device (automatic blood pressure monitor SEM-1, Omron Healthcare Company, Singapore). Body mass was measured wearing light clothing and without shoes using Tanita BC 418MA (Tanita Corporation, Arlington Heights, IL, USA). Body fat mass, fat-free mass, total body water, and phase angle were measured in a lying position after a 10 min rest using a bioelectric impedance analyzer (BIA) Bodystat Quadscan 4000 (Bodystat Ltd., Douglas, Isle of Man, British Isles).

### 2.4. Serum Biomarkers

Venous blood samples were collected in 5 mL serum vacuum blood collection tubes in the morning after anthropometric measurements. Serum samples were prepared after clot formation by full blood centrifugation at 2000 rpm for 10 min. Serum aliquots were immediately frozen and stored at −80 °C. Serum glucose, triacylglycerol (TAG), total cholesterol, low-density lipoprotein (LDL), high-density lipoprotein (HDL), iron, aspartate transaminase (AST), total bilirubin, and C-reactive protein (CRP) levels were measured using Cobas reagents on a Cobas c111 analyzer (Roche, Basel, Switzerland). Serum lipopolysaccharide binding protein (LBP), interleukin-6 (IL-6), and tumor necrosis factor-α (TNF-α) levels were determined in duplicate on a microplate reader (Tecan, Mannedorf, Switzerland) using human ELISA kits (BioVendor, Brno, Czech Republic) for LBP (Cat. No. RD191513100R), IL-6 (Cat. No. RD194015200R), and TNF-α (Cat. No. RAF145R).

### 2.5. Gut Microbiota Composition

Gut microbiota composition was analyzed as described previously [11]. Briefly, DNA was extracted from the frozen stool samples using the commercial QIAamp DNA Stool Mini Kit (Qiagen N. V., Venlo, The Netherlands) following the manufacturer’s instructions. Concentration of DNA was quantified with fluorometer Qubit^®^ 3.0 and Qubit^TM^ dsDNA BR Assay kit (Thermo Fisher Scientific, Hillsboro, OR, USA). The hypervariable region V4 of the 16S rRNA gene was amplified with fusion primers that produced a barcoded sequencing library. Primer 806R contained the sequence of the P1 adapter at its 5′ end, while primer 515Fcontained the sequence of the A adapter, barcode, and linker upstream of the target specific sequence. Each sample was amplified in triplicate. The negative control was prepared for the PCR reaction and sequenced. The PCR reaction mixture and temperature profile were set as described in the Earth Microbiome Project [12]. DNA in pooled triplicate PCR reactions from each sample was measured using the Ion Quantitation Library Kit (Thermo Fisher Scientific, Vilnius, Lithuania). The same amount of DNA from the amplicons was pooled to form the final pool and purified using Agencourt AMPure XP beads (Beckman Coulter, Brea, CA, USA), with a bead to DNA ratio of 0.7:1. The concentration of final pooled amplicon library was determined with the Agilent 2100 Bioanalyzer using the High Sensitivity DNA Assay Kit (Agilent Technologies, Santa Clara, CA, USA). The template for sequencing on the Ion GeneStudio S5^TM^ System was prepared using the Ion 520^TM^ & Ion 530^TM^ Kit-OT2. Samples were sequenced on three Ion 530^TM^ chips (Thermo Fisher Scientific, Santa Clara, CA, USA). Fastq files from each run were imported into QIIME2 v.2021.8 [13]. Cutadapt (qiime cutadapt trim-single) was used to remove primers, and only amplicons with trimmed forward and reverse primers were retained for further analysis. DADA2 [14] (qiime dada2 denois-pyro plugin) was used for denoising and determining amplicon sequence variants (ASVs) using the following arguments: –p-trim-left 0 and –p-trunc-len 0 (resulting in a final set of full-length V4 region sequences). Feature tables and ASVs of samples from different runs were merged using the feature-table merge and merge-seqs plugins. Taxonomy classification was performed with the plugin classify-sklearn. The amplicon-region-specific naive Bayes classifier was trained based on the SILVA reference database, release 138.1, with representative sequences at 99% identity [15]. The reference database was prepared with the RESCRIPt QIIME 2 plugin [16]. The number of reads per sample was normalized to 30,000. Bacterial phyla, families, and genera that were present in at least 10% of the subjects were analyzed. Gut microbiota α-diversity (at the species level) was calculated using the Shannon index.

### 2.6. Questionnaires

#### 2.6.1. Lifestyle Questionnaire

The online lifestyle questionnaire consisted of 9 demographical questions (age, gender, family status, education, socioeconomic status), 17 questions about health family history (diseases, allergies, use of medications and antibiotics, menstruation), 10 questions about sleep and work schedule, 12 questions about the perceived quality of life, 6 questions about substance use (alcohol, smoking, psychoactive substance use), 6 questions about hunger and fullness, 5 questions about factors that could influence gut microbiota composition (mode of birth, having been breastfed, type of environment growing up, and growing up and living with pets), and 6 validated psychological questionnaires: The State-Trait Anxiety Inventory (STAIX-1), The Centre for Epidemiologic Studies Depression Scale (CES-D), The Positive and Negative Affect Schedule (PANAS), A Measure Instrument for Orthorexia Nervosa (ORTO-15), Body Dissatisfaction subscale from the Eating Disorders Inventory-2 (EDI-2) (BD), and Binge Eating (BE). STAIX-1 [17] was used to evaluate the state of anxiety in adults. It contains 20 items and is scored on a 4-point Likert scale; a higher score implies higher anxiety. CES-D [18] was used to measure symptoms associated with depression. It includes 20 items, through which factors such as sleep, appetite, and loneliness are evaluated on a 4-point scale. A higher score indicates a more depressed mood. PANAS was used to determine subjective mood [19]. It measures positive and negative affect and includes 20 adjectives, where subjects indicate to which extent they feel a certain way. Positive affect refers to the extent to which a person feels enthusiastic, active, or alert, and negative affect includes mood states such as anger, contempt, disgust, fear, and nervousness. The ORTO-15 questionnaire [20] was used to measure eating behaviors associated with orthorexia nervosa. It consists of 15 items, through which a subject’s behavior related to the selection, shopping, preparation, and consumption of healthy food is assessed. The questionnaire measures three fundamental components of orthorexia nervosa on a 4-point Likert scale: cognitive–rational, clinical, and the emotional eating component. Items that reflect problematic eating behavior or a tendency to orthorexia are rated as 1, and items that represent normal eating behavior as 4; a higher score represents normal eating behavior. BD [21] consists of ten items assessing how satisfied or dissatisfied an individual is with both overall body shape and the size or shape of specific body parts. Responses are rated on a 5-point Likert scale from 0 to 4; a higher score indicates the highest body dissatisfaction. BE was evaluated by how frequently the subjects rapidly consumed an excessive amount of food in the last week. It includes 10 items that were developed on the basis of the definition in the Diagnostic and Statistical Manual of Mental Disorders [22] of binge eating and the literature in this field [23]. The items are rated 0 or 1; a higher score represents a more frequent occurrence of symptomatology associated with binge eating episodes.

#### 2.6.2. Gastrointestinal Symptoms and Stool Consistency

The frequency of gastrointestinal (GI) symptoms was determined using the subjective Gastrointestinal Symptom Rating Scale (GSRS). Subjects reported the frequency (0—never; 1—hardly ever; 2—sometimes; 3—many times) and intensity (0—none; 1—light; 2—moderate; 3—severe) of nausea, bloating, borborygmi, abdominal pain, flatulence, and heartburn [24]. The subjects were asked about their bowel movement habits, and a Bristol Stool Form Scale (BSFS) was used to subjectively determine stool consistency. The scale consists of 7 types of stool: type 1 represents stool in separate hard lumps, similar to nuts; type 2: sausage-shaped stool, but lumpy; type 3: stool similar to a sausage, but with cracks on its surface; type 4: stool similar to a sausage or snake, smooth, and soft; type 5: stool in soft blobs with clear cut edges; type 6: stool in fluffy pieces with ragged edges; type 7: watery stool, entirely liquid. Types 1 and 2 indicate constipation, whereas 6 and 7 indicate diarrhea. Types 3–5 indicate normal stool consistency [25].

#### 2.6.3. Physical Activity

To determine physical activity, the International Physical Activity Questionnaire (IPAQ) was used. The questionnaire serves to calculate the physical-activity-induced energy expenditure and consists of work-related physical activity, transport-related activity, and activity during leisure time. Data from the duration and intensity of physical activity were used to determine daily energy expenditure in metabolic equivalent of task (MET) [26].

### 2.7. Dietary Intake and Adherence to Mediterranean Diet

The subjects recorded their dietary intake for 3 days using a food diary. They were instructed to weigh foods and beverages before consumption, to weigh any leftovers, and to include food labels and recipes, where applicable. They were also asked to report all dietary supplements taken that day and in general. Dietary data from the food diary were analyzed using the Open Platform for Clinical Nutrition (OPEN), accessible through the website https://opkp.si/, accessed on 10 November 2022. Dietary supplements were calculated and summed to the total daily intake. Adherence to Mediterranean diet was determined using The Mediterranean Diet Adherence Score (MEDAS). It consists of 14 questions that are scored 0 or 1; 12 are related to the food intake frequency and 2 to the food intake habits that are characteristic of the Mediterranean diet. The final score ranged from 0 to 14 [27].

### 2.8. Statistical Analysis

Statistical analysis was performed using IBM SPSS Statistics, version 26.0 (IBM Corp., Armonk, NY, USA). The normality of data distribution was evaluated using the Shapiro–Wilk test. Descriptive variables are expressed as means (M) and standard deviations (SD) for continuous variables, and discrete variables are reported as the frequency (%) of subjects. The chi-squared test was used for categorical variables. Groups of subjects with four dietary patterns were compared using one-way ANOVA or Kruskal–Wallis test, and Tukey or Bonferroni post hoc test. Pearson’s or Spearman’s correlation was used to investigate associations between dietary intake and gut microbiota composition. *p*-values < 0.05 were considered statistically significant.

### 2.9. Visualization of High-Dimensional Dietary Data

The t-distributed stochastic neighbor embedding (t-SNE) was used to observe the distribution between subjects with distinct dietary patterns. It is a nonlinear dimensionality reduction technique that is used to visualize high-dimensional dietary data. The consolidation of the intake of protein (total, animal, plant), carbohydrates (total, sugars, free sugars, dietary fiber), and fats (total, SFA, ω-3 FA, ω-6 FA, MUFA, PUFA) into principle components (relative to total daily intake) was used for visualization. The t-SNE algorithm embeds high-dimensional points into low dimensions in such a way that similarities between points are reflected and distant (near) points in high-dimensional space correspond to distant (near) embedded low-dimensional points. The basic steps of the t-SNE algorithm are as follows: (i) computing the pairwise distance between all points in the high-dimensional space, (ii) computing a standard deviation for each high-dimensional point such that the perplexity of each point is at a predetermined level, (iii) computing the similarity matrix, (iv) creating an initial set of low-dimensional points, and then minimizing the Kullback–Leibler divergence between a Gaussian distribution in the high-dimensional space and a t-distribution in the low-dimensional space. The analysis was conducted in MATLAB 2020A.

### 2.10. Cluster Analysis and General Predictors of Gut Microbiota Composition

Clustering analysis of the microbiota dataset at the genus level was performed using an unsupervised technique called k-means clustering. The k-means algorithm clusters data into similar subsets, minimizing the distances within a cluster and maximizing the distance between different clusters. In the present study, the clustering criterion was the sum of squared Euclidean distances between each data point x_i_ and the centroid m_k_ (cluster center) of the subset c_k_ containing x_i_. We used the elbow method to determine the optimal number of clusters.

After the clustering analysis of the microbiota data, we built a model to predict the clustering classes of the microbiota. One hundred and ninety-nine features were initially used to build the model. We used the k-nearest neighbor classifier to build a model and predict the microbiota classes based on the variables. The k-nearest neighbor classifier finds the k-nearest neighbors whose classes are known and then assigns the classification label to a new input. The input is assigned to the class with which it shares the nearest neighbors. For this particular study, we used cosine distance to calculate the similarities between data points and two nearest neighbors. In other words, our model was a 2-nearest neighbor classifier using the cosine of the included angle between variables as the distance metric.

The *sequentialfs* function is a MATLAB function and part of the Statistics and Machine Learning Toolbox. The *sequentialfs* function selects features sequentially based on a user-defined criterion. After calculating the mean criterion values for each candidate feature subset, *sequentialfs* selects the candidate feature subset that minimizes the mean criterion value. This process continues until adding or removing more features no longer decreases the criterion. There are generally two options for sequential search: forward and backward. We used the backward search, where the search starts with all 199 variables and an algorithm sequentially removes features until the criterion decreases. The criterion used in this study was classification error. Thus, at each step of feature selection, a model is created using the k-nearest neighbor method, which is validated using the leave-one-out procedure. This process is repeated until the criterion (classification error) decreases.

The predictive power of the k-nearest-neighbor classifier was tested by leave-one-out cross-validation, a special case of k-fold cross-validation where k is equal to the number of data points in the dataset. Leave-one-out cross-validation uses the entire dataset to build the model, except for one data point. The prediction is made for a single point that is excluded from the training set. The predicted value is then compared to the true value for validation purposes. The entire process is repeated k times, where k is the number of data points in the dataset.

## 3. Results

### 3.1. Characteristics of Subjects with Distinct Dietary Patterns

As shown in Table 1, subjects with four distinct dietary patterns (O, V, VE, LCHF) did not significantly differ in age, gender, anthropometric measurements, education, or socioeconomic status. Regarding the relationship between dietary patterns and lifestyle factors that could influence the gut microbiota composition, a statistically significant relationship between groups was observed only in the type of environment growing up (*χ*^2^ (3) = 10.965, *p* = 0.012), and growing up with pets (*χ*^2^ (3) = 13.732, *p* = 0.003). The majority of V (75.0%) and only 29.2% of O grew up in a rural environment. The same was observed for pets, as 91.7% V grew up with pets, compared to only 41.7% of O. No other statistically significant relationships were observed between dietary pattern and lifestyle factors.

### 3.2. Serum Biomarkers in Subjects with Distinct Dietary Patterns

Despite no major differences in lifestyle factors, we were interested in differences in serum biomarkers between subjects with four distinct dietary patterns that are presented in Table 2. Statistically significant differences between the four groups were observed for serum cholesterol (*χ*^2^ (3) = 24.550, *p* < 0.001), HDL (*F* (3,85) = 6.176, *p* = 0.001), and LDL (*χ*^2^ (3) = 18.727, *p* < 0.001) levels that were the highest in LCHF and lowest in V. The average cholesterol and LDL levels in LCHF exceeded Slovenian reference values (LCHF–all other diets *p* < 0.05). All groups had, on average, adequate HDL levels (LCHF–V *p* < 0.001). Serum iron levels were the highest in O and lowest in LCHF (O–LCHF and O–VE *p* < 0.01), and a statistically significant difference was observed between the four groups (*F* (3,85) = 7.756, *p* < 0.001). No statistically significant differences between groups were observed in serum glucose levels, inflammatory profile, and other serum biomarkers.

### 3.3. Dietary Intake in Subjects with Distinct Dietary Patterns

The analysis of dietary intake is presented in Table 3. As expected, the four dietary patterns differed significantly in the intake of protein (all types), carbohydrates (total, sugar, dietary fiber), and fats (total, SFA, and MUFA) (*p* < 0.001). The intake of ω-3 FA (*p* = 0.008) and ω-6 PUFA (*p* = 0.010) was also significantly different, and the same was found for EPA, DHA, and cholesterol intake (*p* < 0.001). V was the only group in which the average intake of all macronutrients was in line with the recommended dietary intake (RDI) for the Slovenian population (Table 3).

Total and animal protein intake was the highest in LCHF and lowest in V, and the contrary occurred for plant protein (LCHF–all other diets and O–V *p* < 0.05), carbohydrates, and dietary fiber (LCHF–all other diets and O–V *p* < 0.05). Only V and VE reached the RDI for dietary fiber (>30 g). The intake of free sugars was the lowest in LCHF (LCHF–VE and LCHF–O *p* < 0.001, V–O *p* = 0.035), but no group exceeded the RDI. The intake of fats, SFA, and cholesterol was the highest in LCHF and lowest in V (LCHF–all other diets *p* < 0.001, V–VE and O–V *p* < 0.05), and the same was true for MUFA (LCHF–all other diets *p* < 0.001). The intake of ω-3 (LCHF–all other diets *p* < 0.05) and ω-6 (LCHF–O *p* = 0.008) PUFA was adequate in all groups and was the highest in LCHF (Table 3).

The intake of micronutrients represents the sum of micronutrient intake from diet and from dietary supplements (not described in Table 3). The intake of calcium was the lowest in V (755.5 mg), and did not reach the RDI (1000 mg); however, the difference between the groups was not significant. The same was true for vitamin D, where only VE (25.5 µg) reached the RDI (20 µg). Statistically significant differences between the four groups were observed in the intake of biotin, folate, vitamin B12, copper, manganese (*p* < 0.001), selenium, riboflavin, pantothenic acid, α- and β-carotene (*p* < 0.01), vitamin E, vitamin K, and vitamin C (*p* < 0.05). V had the highest intakes of α- and β-carotene, vitamin K (LCHF–V *p* < 0.05), copper (V–LCHF and VE–LCHF *p* < 0.01), vitamin E (LCHF–O *p* = 0.017), vitamin C (O–V *p* = 0.007), and folate (O–V *p* = 0.002, LCHF–V *p* = 0.020). Due to dietary supplements, the intake of vitamin B12 was also the highest in V (VE–V and O–V *p* < 0.01, VE–LCHF and O–LCHF *p* < 0.05). On the other hand, LCHF had the highest intake of biotin, pantothenic acid, manganese, selenium (LCHF–V and LCHF–VE *p* < 0.01), and riboflavin (LCHF–V *p* = 0.002).

Along with other dietary supplements, we also analyzed the intake of probiotics and found a statistically significant difference between the four groups (*p* = 0.021). The intake of probiotics was the highest in V (29.2%), whereas none in the LCHF reported taking probiotics. The subjects also reported taking other dietary supplements (not described in Table 3); the most frequent ones were collagen, algae, and methylsulfonylmethane (MSM) in all diet groups.

In addition to the food diary analysis, we assessed the subjects’ adherence to a Mediterranean diet that was significantly different between groups (*F* (3,85) = 10.502, *p* < 0.001), and was the highest in V and lowest in LCHF (V–LCHF *p* < 0.001, O–V and VE–LCHF *p* < 0.01) (Table 3).

**Figure 2:**
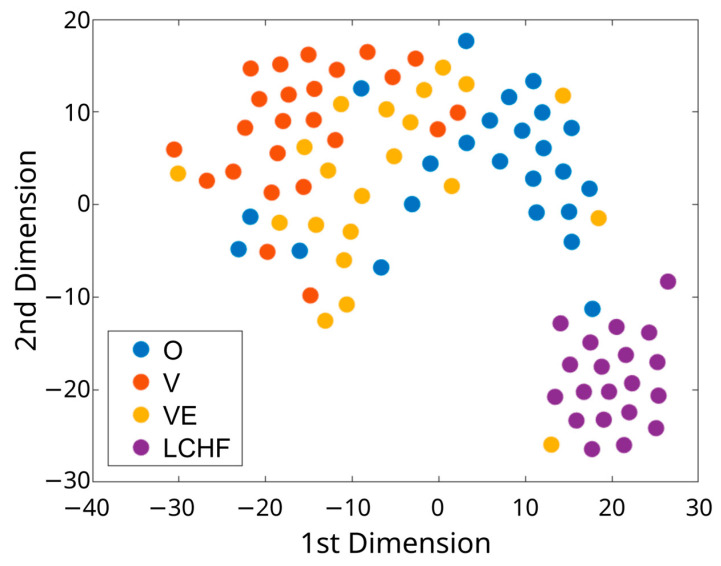
The t-distributed stochastic neighbor embedding (t-SNE) plot for the dietary dataset of subjects with distinct dietary patterns (O, V, VE, LCHF). O—omnivorous; V—vegan; VE—vegetarian; LCHF—low-carbohydrate, high-fat.

Many differences in dietary intake were observed between the four groups, especially with LCHF, which was distinctively different from other diet groups. The t-distributed stochastic neighbor embedding plot revealed a somewhat clear separation of the dietary intake between LCHF, O, and V. LCHF was the most distinct diet group compared to other three groups, whereas VE was a more heterogenous group and was distributed mainly between O and V (Figure 2).

### 3.4. GI Symptoms and Gut Microbiota Composition in Subjects with Distinct Dietary Patterns

In addition to dietary intake, we were interested in differences in stool consistency and GI symptoms between subjects with four distinct dietary patterns (O, V, VE, LCHF); these are presented in Table 4. The subjects adhering to an LCHF diet reported having fewer GI symptoms. The frequency of bloating between the four groups was significantly different (*χ*^2^ (3) = 10.029, *p* = 0.018), and the same was the case for flatulence (*χ*^2^ (3) = 14.581, *p* = 0.002). The frequency of flatulence was the lowest in LCHF and highest in V (LCHF–V *p* = 0.001, LCHF–O *p* = 0.022) (Table 4). For stool consistency, a statistically significant difference was observed between the four groups (*χ*^2^ (3) = 9.989, *p* = 0.019), with V having the loosest stools and LCHF the hardest (LCHF–V *p* = 0.020) (Table 4).

Our primary research focus was on differences in gut microbiota composition in subjects with four distinct dietary patterns; these are presented in Table 5. Firstly, we compared the gut bacteria at the phylum level. Bacteroidota and Firmicutes were the predominant phyla and represented more than 90% of the whole gut microbiota in all dietary patterns. A significant difference in relative abundance of the phylum Actinobacteria was observed between the four groups (*χ*^2^ (3) = 22.613, *p* < 0.001), with LCHF having the lowest abundances (LCHF–all other diets *p* < 0.05). On the contrary, LCHF had the highest abundances of Desulfobacterota (V–LCHF and VE–LCHF *p* < 0.05), which was significantly different between the four groups (*χ*^2^ (3) = 10.024, *p* = 0.018). A significant difference in the relative abundance of Verrucomicrobiota was also observed (*χ*^2^ (3) = 9.804, *p* = 0.020); O had the highest abundances and V the lowest (O–V *p* = 0.016). Interestingly, although not significant, the lowest α-diversity of gut microbiota was detected in V (Table 5).

**Figure 3:**
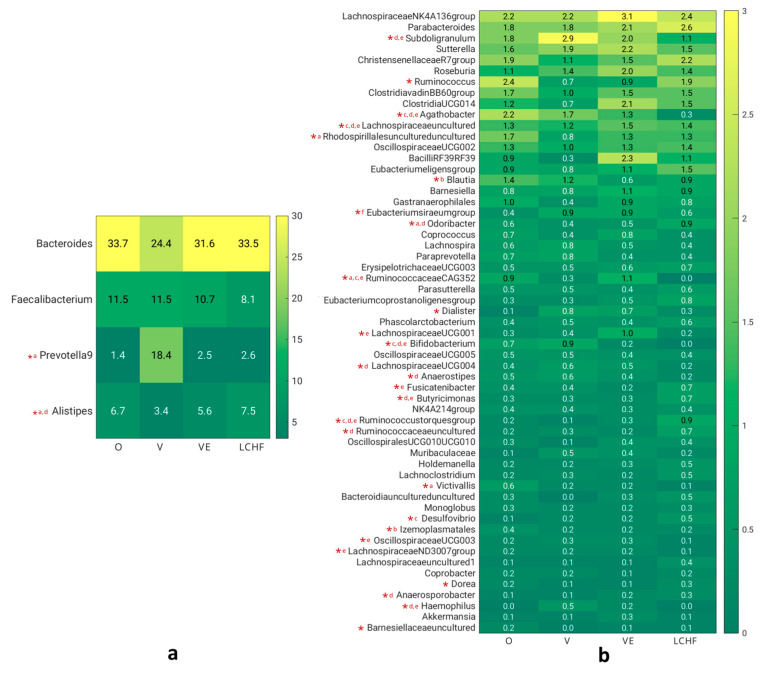
(**a**) Relative abundance of most represented gut microbial genera. (**b**) Relative abundance of less represented gut microbial genera. O—omnivorous; V—vegan; VE—vegetarian; LCHF—low-carbohydrate, high-fat; ^a^—O–V; ^b^—O–VE; ^c^—O–LCHF; ^d^—V–LCHF; ^e^—VE–LCHF; ^f^—V–VE (post hoc tests); *—statistically significant difference (ANOVA).

Secondly, we compared the gut bacteria, present in at least 10% of the subjects, at the genus level (Figure 3). The four most represented genera were *Bacteroides*, *Faecalibacterium*, *Prevotella 9*, and *Alistipes* (Figure 3a). A statistically significant difference between the four groups was found for the relative abundance of *Prevotella 9* (*χ*^2^ (3) = 9.831, *p* = 0.020), which was most represented in V, and *Alistipes* (*χ*^2^ (3) = 11.167, *p* = 0.011), which was most represented in LCHF and least in V.

Some significant differences between the four groups were also observed for less-represented genera (Figure 3b). Significant differences between the four groups (Kruskal–Wallis test) and significant differences between specific diet groups compared with a post hoc test are marked in Figure 3. The relative abundances of *Bifidobacterium*, *Haemophilus* (*p* < 0.001 for both), *Lachnospiraceae UCG-004* (*p* = 0.003), *Subdoligranulum,* and *Anaerostipes* (*p* = 0.012 for both) were the highest in V and lowest in LCHF. The genera that were predominant in VE and least represented in LCHF were *Ruminococcaceae CAG-352* (*p* < 0.001), *Lachnospiraceae UCG-001* (*p* = 0.013), and *Oscillospiraceae UCG-003* (*p* = 0.030), whereas *Agathobacter*, *Lachnospiraceae ND3007,* and *Victivallis* (*p* = 0.020 for all) were least represented in LCHF and most in O. Some other genera that were predominant in O were *Ruminococcus* (*p* = 0.034), *Rhodospirillales uncultured* (*p* = 0.005), *Blautia* (*p* = 0.009), and *Izemoplasmatales* (*p* = 0.011). On the contrary, the genera that were predominant in LCHF were *Ruminococcus torques*, *Lachnospiraceae uncultured* (*p* < 0.001 for all), *Odoribacter* (*p* = 0.001), *Butyricimonas* (*p* = 0.003), *Ruminococcaceae uncultured* (*p* = 0.008), *Fusicatenibacter* (*p* = 0.034), *Desulfovibrio* (*p* = 0.026), and *Anaerosporobacter* (*p* = 0.014).

**Figure 4:**
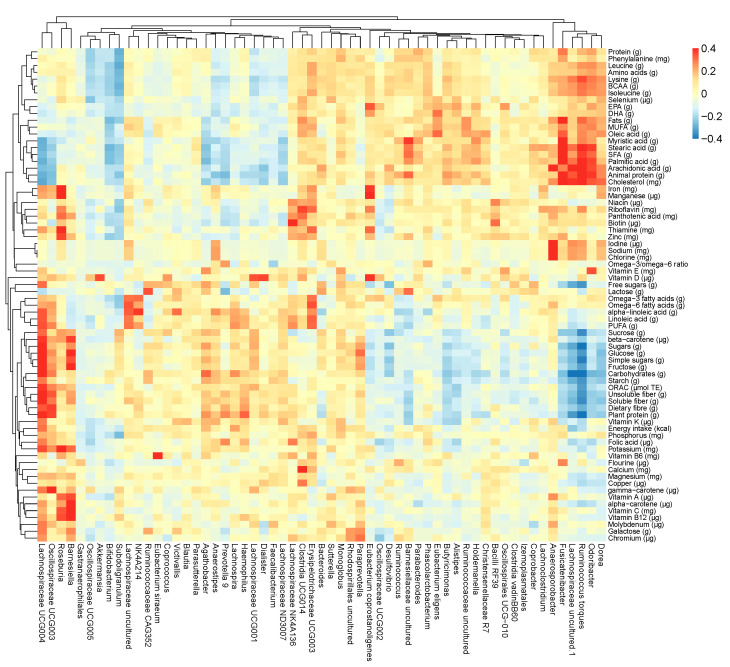
Heatmap of Spearman’s correlation coefficient between dietary intake (on the right side) and gut microbial genera (at the bottom).

In addition to the analysis of dietary intake and gut microbiota composition (at the genus level), we focused on the relationship between the two. Some significant correlations were observed and are presented in Figure 4. Positive correlations are marked with red color, whereas negative are marked with blue color. Many correlations were observed for the intake of macronutrients, and less for micronutrients. Positive (ρ ≥ 0.4) or negative (ρ ≤ −0.4) correlations that are at least moderate are described.

The intake of carbohydrates and plant protein was positively correlated with the relative abundance of *Lachnospiraceae UCG-004*, *Agathobacter*, *Haemophilus*, *Bifidobacterium*, and *Anaerostipes*, and negatively with *Lachnospiraceae uncultured* and *Ruminococcus torques* (*p* < 0.001 for all). Similarly, the intake of dietary fiber was positively correlated with *Lachnospiraceae UCG-004* and *Haemophilus*, and negatively with *Ruminococcus torques* (*p* < 0.001 for all).

The intake of fats and SFA was positively correlated with the relative abundance of *Ruminococcaceae uncultured*, *Ruminococcus torques*, *Anaerosporobacter*, and *Odoribacter*, and negatively with *Bifidobacterium* (*p* < 0.001 for all). Likewise, the intake of animal protein was positively correlated with the relative abundance of *Butyricimonas*, *Lachnospiraceae uncultured*, *Ruminococcus torques*, *Odoribacter*, *Ruminococcaceae uncultured*, *Barnesiellaceae uncultured*, *Rhodospirillales uncultured*, *Anaerosporobacter* (*p* < 0.001 for all), and *Alistipes* (*p* = 0.007), and negatively with *Bifidobacterium, Haemophilus,* and *Lachnospiraceae UCG-004* (*p* < 0.001 for all).

For micronutrients, a negative correlation was observed between the intake of pantothenic acid and biotin, and the relative abundance of *Bifidobacterium* (*p* < 0.001). The intake of copper and manganese was positively correlated with the relative abundance of *Lachnospiraceae UCG-004* (*p* < 0.001), and negatively with *Ruminococcus torques* (*p* < 0.01).

### 3.5. Cluster Analysis for Gut Microbiota Composition

**Figure 5:**
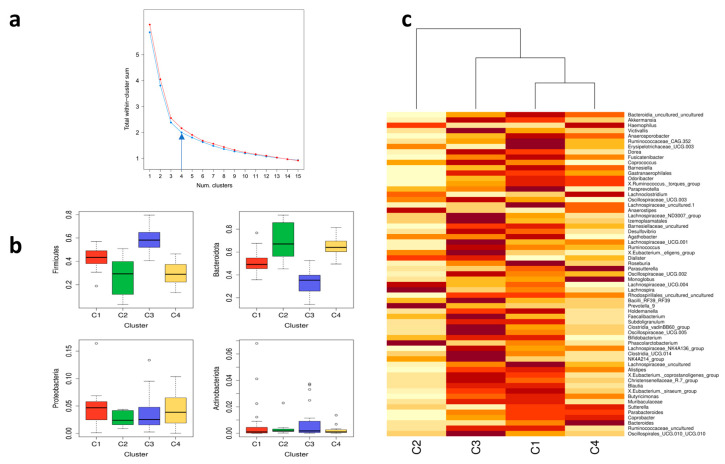
Elbow, box plot, and heatmap of gut microbiota composition in subjects (*n* = 89). (**a**) Total within-cluster sum of squares versus number of clusters computed by k-means clustering (red color for the family level and blue color for the genus level). (**b**) The relative abundance of the most abundant gut microbial phyla (Firmicutes, Bacteriodota, Proteobacteria, and Actinobacteria) in four clusters (C1, C2, C3, C4). (**c**) A heatmap of the relative abundance of different genera in the gut microbial community in four clusters (C1, C2, C3, C4).

An essential research question of microbiome study is to determine whether the microbiota can be stratified into subgroups, and if so, how many groups are there, and how to interpret the strata. We were especially interested in whether gut microbiota (at the genus level) could be a useful indicator of a long-term dietary pattern and if we could determine an individual’s diet solely based on gut microbiota composition. As illustrated in Figure 5a, hierarchical clustering (at the genus and at the family level) revealed an elbow at k = 4, suggesting that the dataset can be organized into four clusters. C2 (*n* = 8) was constituted only of V (100%), whereas C1, C3, and C4 included representatives of all dietary patterns, regardless of the clustering being performed at the genus or at the family level. As a consequence, we decided to focus on the genus level. In C1 (*n* = 28), the most predominant was LCHF (32.2%), then VE (25%), O, and V (both 21.4%). In C3 (*n* = 33), the predominant was O (33.3%), followed by VE (27.3%), V (21.2%), and LCHF (18.2%), and in C4 (*n* = 20) it was O (35.0%), then LCHF, VE (both 25.0%), and V (15.0%).

For phyla, C1 was most abundant in phylum Proteobacteria, C2 in Bacteroidota, C3 in Actinobacteria and Firmicutes, and C4 in Bacteriodota and Proteobacteria (Figure 5b). Hierarchical clustering revealed that the most similar clusters were C1 and C4, whereas C3 was somewhat similar to C1 and C4. The most distinctly different cluster from others was C2, which was constituted only of V (Figure 5c).

For genera, C1 was most abundant in genera *Alistipes*, *Roseburia*, *Agathobacter*, *Lachnospiraceae uncultured*, and *Barnesiella*; C2 in genera *Prevotella 9*, *Lachnospira*, *Phascolarctobacterium*, and *Anaerostipes*; C3 in genera *Faecalibacterium*, *Lachnospiraceae NK4A136*, *Clostridia vadinBB60*, *Bacilli RF39*, *Christensenellaceae R-7*, and *Clostridia UCG-014*; and C4 in genera *Bacteroides*, *Parasutterella*, and *Monoglobus* (Figure 5c). The hierarchical clustering revealed that gut microbiota composition at the genus level is therefore not a useful indicator of a subject’s dietary pattern, with the exception of a high abundance of the genus *Prevotella 9*, which indicates a V diet. However, an individual could be adhering to a V diet and not have this specific type of gut microbiota composition. O were classified in C1, C3, and C4, with C3 and C4 being more likely. Similar was the case for subjects following an LCHF diet; however, they were more likely to be classified in C1 and C4. On the other hand, VE were classified in all three clusters almost equally.

### 3.6. Variable Selection

**Figure 6:**
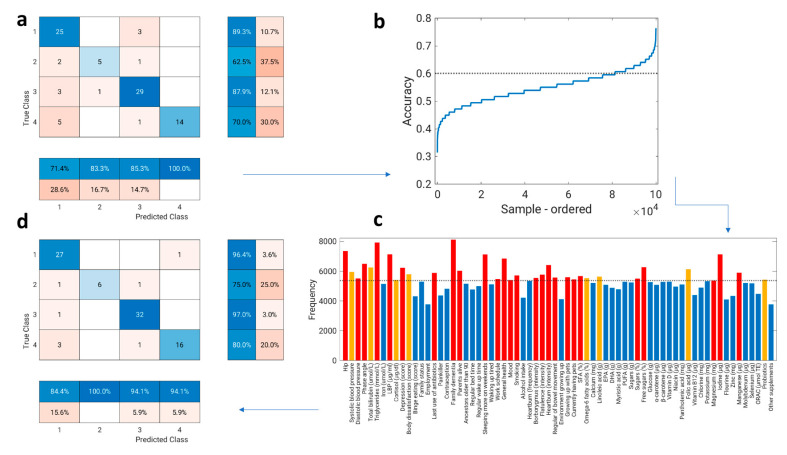
Flow chart of variable selection. (**a**) The confusion matrix of k-nearest neighbor classifier model where 68 predictors were included (82% accuracy). (**b**) The accuracy for all 100,000 random datasets (from 68 predictors, we randomly selected 20 predictors that we used to build a model and calculate the accuracy of a model; we repeated this process 100,000 times; at each step, a different random set of 20 variables was selected). (**c**) The frequency of features from the sets that provide an accuracy of 60% or more (predictors whose frequency corresponded to the 50th percentile are colored red or orange; those below the 50th percentile are colored blue). (**d**) The confusion matrix of the k-nearest neighbor classifier model where 26 predictors were included (91% accuracy). The final predictors (26) are colored red in (**c**).

With the exception of C2 with the predominance of *Prevotella 9* that was constituted only of V, we could not determine an individual’s dietary pattern solely based on gut microbiota composition. Thus, we further investigated which specific nutrients or other lifestyle factors, not related to dietary intake, predict specific clusters. First, we started feature selection with all 199 variables (supplementary document) using sequential feature selection—*sequentialfs*. The MATLAB *sequentialfs* function excluded 131 variables. Using 68 remaining variables as input to the k-nearest neighbor classifier model resulted in 82% accuracy (Figure 6a). Interpretation of 68 predictors is quite difficult, so we took further steps to reduce the number of variables and maintain predictive power. Since an exhaustive comparison of criterion value is usually not feasible for all subsets of all possible combinations, we used a random selection algorithm to reduce the number of predictive variables. From 68 predictors, we randomly selected 20 predictors that we used to build a model and calculate its accuracy. We repeated this process 100,000 times. At each step, a different random set of 20 variables was selected. Figure 6b shows the accuracy for all 100,000 random sets. Note that the accuracy is sorted from highest to lowest. We can see that a few sets give very high (>70%) and a few give very low (<40%) accuracy. Further, we selected only the sets that provide accuracy above 60%. The frequency of predictors from the sets that provide an accuracy of 60% or more is shown in Figure 6c. It is clear that some features, such as family history of dementia and serum TAG levels, have higher frequencies than employment and alcohol intake, for example. Further, we selected predictors whose frequency corresponded to the 50th percentile. Thus, the new smaller subset contained only 34 variables (Figure 6c, red and orange bars). Finally, we performed sequential feature selection again for the subset that contained 34 variables. The *sequentialfs* function excluded an additional eight features. The final set contained 26 variables and achieved 91% accuracy (Figure 6d, confusion matrix). The final set of variables was, thus, about 2.5 times smaller than the first subset (68), but the accuracy actually increased slightly, from 82 to 91%.

### 3.7. Predictors

Significant gut microbiota composition cluster predictors (26) are presented in Table 6. With these model predictors we can very accurately predict subjects’ classification in C1 and C3 (96.4% and 97%), whereas we can somewhat less, but still very accurately, predict classification in C2 (75%). The most important gut microbiota composition predictors were from the following categories: anthropometric measurements, serum biomarkers, lifestyle factors, GI symptoms, psychological factors, and specific nutrients intake. We further analyzed if any significant relationship exists between clusters and model predictors. A statistically significant relationship between clusters and predictors was observed for hip circumference (*χ*^2^ (3) = 11.842, *p* = 0.008), phase angle (*χ*^2^ (3) = 8.758, *p* = 0.033), work schedule (*χ*^2^ (3) = 20.912, *p* = 0.013), having alive parents (*χ*^2^ (3) = 14.553, *p* = 0.024), growing up with pets (*χ*^2^ (3) = 10.655, *p* = 0.014), and the intake of SFA (*χ*^2^ (3) = 11.809, *p* = 0.008) and iodine (*χ*^2^ (3) = 12.612, *p* = 0.006). Subjects in C1 had the highest hip circumference (C1–C4 *p* = 0.004), and C4 the lowest. C2 was the most distinct group, with the highest phase angle (C2–C3 *p* = 0.038), the most flexible work schedule, and was the only group where all subjects grew up with pets and had both parents alive. The intake of SFA (C2–all other clusters *p* < 0.05) and iodine (C2–C3 and C2–C4 *p* < 0.05) was also the lowest in C2.

## 4. Discussion

To investigate the relationship between distinct dietary patterns and gut microbiota composition, a cross-sectional study in subjects adhering to omnivorous (O), vegetarian (VE), vegan (V), and low-carbohydrate, high-fat (LCHF) diet was performed. The subjects were equally distributed between groups, were from the same geographical location, and did not differ in age, gender, anthropometric measurements, education, or socioeconomic status, which are factors that can significantly influence gut microbiota composition [28]. A statistically significant relationship was observed between dietary pattern and the type of environment growing up and growing up with pets, as the majority of V and the minority of O grew up in a rural environment with pets. Similarly, a recent study showed that individuals who grew up around a variety of pets were more likely to engage in greater levels of veganism [29]. Living in a rural environment could influence environmental consciousness, and it has been observed that the progression from O diet to VE and V diets is associated with increased environmental sustainability [30].

Despite no major differences in lifestyle factors, we looked for any differences in serum biomarkers between the four groups. We were especially interested in lipid profile and inflammatory status, factors that have been favorably associated with plant-based diets [31]. Statistically significant differences between the groups were observed for serum cholesterol, LDL, and HDL levels that were the highest in LCHF and lowest in V. The same increase in LDL after adhering to an LCHF diet was observed in other studies, at least in short-term studies [32,33,34], whereas long-term studies are lacking. However, a meta-analysis showed no significant differences in LDL after 6, 12, and 24 months of an LCHF diet [35]. Nevertheless, it is important to interpret these results with caution, as the majority of studies use LCHF diets as a weight-loss tool in subjects with obesity, whereas the subjects in our study had a normal body mass which was stable and were not pursuing weight loss.

In addition to serum biomarkers, many differences in dietary intake between the groups were observed. The consolidation of dietary intake into principle components revealed clear separation between the groups. VE was a more heterogenous group compared to others, as some only exclude meat from the diet, whereas others also exclude fish, dairy, or eggs [36]. As expected, the four diet groups differed significantly in the intake of all macronutrients. Total and animal protein intake was the highest in LCHF, which is typical for an LCHF diet [37], and lowest in V, and similar observations were made in studies that compared V with VE and O [38,39]. As expected, the intake of fats, SFA, MUFA, and cholesterol was the highest in LCHF and lowest in V, and the contrary occurred for the intake of plant protein, carbohydrates, and dietary fiber. Only V and VE reached the RDI for dietary fiber, similarly to a recent systematic review [40]. The intake of free sugars was the lowest in LCHF, which is typical for an LCHF diet [41]. Many differences in the intake of micronutrients were observed between groups (summed from diet and dietary supplements). V had the highest intakes of α- and β-carotene, vitamin K, copper, vitamin E, vitamin C, folate, and also vitamin B12, due to dietary supplements. Similarly, a higher intake of folate and vitamins C and E was observed in plant-based diets compared to meat-eaters [40]. On the other hand, LCHF had the highest intake of biotin, pantothenic acid, manganese, selenium, and riboflavin. Additionally, we observed a higher adherence to Mediterranean diet in V and VE, and the same was reported in other research [39,42].

Our primary research focus was gut microbiota composition, and we were also interested in differences in stool consistency and GI symptoms between the groups. LCHF reported having fewer GI symptoms, especially flatulence, which was the highest in V. V also had the loosest stools, which was already shown in previous research that observed that consuming more dietary fiber was associated with softer stools [43]. It has been known for a long time that Bacteroidota and Firmicutes are the predominant phyla and represent more than 90% of the whole gut microbiota [7], and we observed the same in all four diet groups. Significant differences in the relative abundance of Actinobacteria, Desulfobacterota, and Verrucomicrobiota were observed between groups. LCHF had the lowest abundance of Actinobacteria and the highest abundance of Desulfobacterota. Similarly, a lower abundance of Actinobacteria was observed in children after a 6-month ketogenic diet [44]; however, long-term studies are lacking. O had the highest abundance of Verrucomicrobiota, whereas V had the lowest. The four predominant genera in all diet groups were *Bacteroides*, *Faecalibacterium*, *Prevotella 9*, and *Alistipes*, and many significant differences were observed between groups. In V, the predominant genera were *Prevotella 9*, *Bifidobacterium*, *Haemophilus*, *Lachnospiraceae UCG-004*, *Subdoligranulum*, and *Anaerostipes*. Similar observations about the association of the genus *Prevotella* with a high intake of carbohydrates, which is typical for a V diet, were already made in previous research [45]. Plant foods high in polyphenols, frequently consumed in V, have been associated with a higher abundance of *Bifidobacterium* [46], and a higher abundance of *Subdoligranulum* in V and VE compared to O was observed previously in the Slovenian population [47]. In VE, the predominant genera were *Ruminococcaceae CAG-352*, *Lachnospiraceae UCG-001*, and *Oscillospiraceae UCG-003*. In O, the predominant genera were *Agathobacter*, *Lachnospiraceae ND3007*, *Victivallis, Ruminococcus*, *Rhodospirillales uncultured*, *Blautia*, and *Izemoplasmatales.* One study observed a higher abundance of *Blautia* in O compared to V [48]. In LCHF, the predominant genera were *Alistipes*, *Ruminococcus torques, Lachnospiraceae uncultured*, *Odoribacter*, *Butyricimonas*, *Ruminococcaceae uncultured*, *Fusicatenibacter*, *Desulfovibrio,* and *Anaerosporobacter*. Similarly, an increase in the abundance of *Alistipes*, *Odoribacter*, *Butyricimonas,* and *Desulfovibrio* and a decrease in the abundance of *Bifidobacterium* was observed in overweight adults after a 4-week LCHF diet designed for weight loss [49]; however, long-term studies in adults with a normal BMI are lacking.

Additionally, we focused on the relationship between dietary intake and gut microbiota composition. The intake of dietary fiber, carbohydrates, and plant protein was positively correlated with *Lachnospiraceae UCG-004* and *Haemophilus*, and the intake of carbohydrates and plant protein was also positively correlated with *Agathobacter*, *Bifidobacterium,* and *Anaerostipes.* Similar to our study, a study in adult men observed an association between dietary fiber intake and *Haemophilus* and *Bifidobacterium* [50], and it is clear that *Bifidobacterium* are able to utilize a diverse range of dietary carbohydrates [51]. Several species of Lachnospiraceae were also associated with dietary fiber and plant protein intake in previous research [52]. In the present study, the intake of fats, SFA, and animal protein was positively correlated with *Ruminococcaceae uncultured*, *Ruminococcus torques*, *Anaerosporobacter*, and *Odoribacter*, and the intake of animal protein was also positively correlated with *Butyricimonas*, *Lachnospiraceae uncultured*, *Barnesiellaceae uncultured*, *Rhodospirillales uncultured*, and *Alistipes*. Similarly, a higher abundance of *Ruminococcaceae uncultured* was observed in subjects with high SFA intake [53]. A higher abundance of *Odoribacter* was observed in mice fed a diet rich in animal protein [54], and *Alistipes* in humans consuming an animal-based diet [55].

After determining the differences between the four diet groups, we were interested in whether gut microbiota at the genus level could be a useful indicator of a long-term dietary pattern. Hierarchical clustering revealed that subjects can be classified in four clusters depending on gut microbiota composition; C1 was most abundant in *Alistipes, Roseburia, Agathobacter*, *Lachnospiraceae uncultured,* and *Barnesiella*; C2 in *Prevotella 9*, *Lachnospira*, *Phascolarctobacterium*, and *Anaerostipes*; C3 in *Faecalibacterium*, *Lachnospiraceae NK4A136*, *Clostridia vadinBB60*, *Bacilli RF39*, *Christensenellaceae R-7*, and *Clostridia UCG-014*; and C4 in *Bacteroides*, *Parasutterella*, and *Monoglobus*. C2 constituted only of V, whereas other clusters were mixed depending on the dietary pattern. Thus, we can conclude that gut microbiota composition at the genus level is not a useful indicator of a subject’s dietary pattern, with the exception of a high abundance of the genus *Prevotella 9*, which indicates a V diet. However, it is important to note that an individual could be following a V diet and not have this specific gut microbiota composition, as V were also classified in C1, C3, and C4. Most subjects following an LCHF diet were classified in C1, which is characterized by a high abundance of Proteobacteria and *Alistipes.* Indeed, diets with a low intake of fiber and a high intake of fats have been shown to increase the abundance of *Alistipes* [55,56], and intake of dietary cholesterol was shown to correlate with Proteobacteria [57]. Most O were classified in C3 and C4, whereas VE was the most heterogenous group and was almost equally classified in C1, C3, and C4.

After this observation, we built a model to explain which lifestyle factors can predict specific gut microbiota composition regardless of dietary pattern. The limitation of the present study is a relatively small sample size. In order to have the groups of subjects adhering to four different dietary patterns homogenous by age, gender, and BMI, we included a sample size of 89 subjects. Subjects adhering to LCHF were particularly hard to recruit, as they needed to be adhering to an LCHF diet for a minimum of 6 months while also having a suitable BMI and keeping a stable body mass for at least 3 months. All of these criteria substantially limited our sample size. Because of the small sample size, the hold-out method and also 10-fold cross-validation were not the right choice to validate our model. Instead, we used the leave-one-out method, which is appropriate for small datasets. A larger dataset would also allow us to perform nested cross-validation and thus optimize the hyperparameters independently, e.g., the similarity distance and the number of nearest neighbors in the case of the k-nearest neighbor classifier and the final set of variables. The choice of these parameters is biased to some extent, since they were not optimized by nested cross-validation, which could lead to an overly optimistic result.

Among anthropometric measurements, significant predictors of gut microbiota composition were hip circumference, phase angle, and diastolic blood pressure. Subjects in C1 had the highest hip circumference, and C4 the lowest, whereas phase angle was the highest in C2. Similarly, it has been observed that anthropometric measurements such as BMI, mid-upper arm, and waist circumference, and waist-to-hip ratio were significantly associated with lower α-diversity and changes in gut microbiota composition [58]. Additionally, one study identified measures of obesity (waist-to-hip ratio, BMI, visceral fat index) as significant gut microbiota composition predictors in healthy adults [59]. It seems that the gut microbiota also plays an important role in the development and pathogenesis of hypertension [60], as hypertension and systolic blood pressure have been inversely associated with α-diversity of gut microbiota [61]. In the present study, subjects in C2, where *Prevotella 9* was a predominant genus, had the most favorable anthropometric measurements.

Significant gut microbiota composition predictors from the group of serum biomarkers were serum levels of TAG and LBP. Similarly, it has been observed that gut microbiota is associated with blood lipids metabolism in healthy adults, independent of age, gender, and genetics [62]. LBP, which is highly correlated with lipopolysaccharide (LPS) levels, has been recognized as a reliable systemic biomarker of intestinal permeability, especially in healthy adults who generally have low concentrations of LPS [63]. Only one study in healthy premenopausal women observed an association between LBP levels and changes in diversity and gut microbiota composition, especially with bacteria that were previously associated with obesity and inflammation, such as *Bacteroides* [64]. In our study, subjects in C1, with a high abundance of Proteobacteria and *Alistipes*, had a worse metabolic profile compared to other clusters, with higher levels of TAG and LBP. Similarly, *Alistipes* has been implicated to play a critical role in inflammation and disease [65], and the same is true for Proteobacteria [66]. Higher abundances of *Alistipes* have also been associated with TAG in children [67].

Many lifestyle factors were identified as significant gut microbiota composition predictors, such as growing up with pets, currently having pets, smoking, sleeping more on weekends, work schedule, last use of antibiotics, family history of dementia, and having alive parents. C2 was the most distinct group, and was the only group where all subjects grew up with pets and had both parents alive, and was also the group where family history of dementia was the most prevalent. On the other hand, in C3, current pet ownership was the most prevalent and the family history of dementia the least prevalent among all clusters. The gut microbiota has been proposed as a determinant of healthy aging, as a higher prevalence of health-associated bacteria, such as *Bifidobacterium* and *Christensenellaceae*, has been associated with longevity [68]. The association between a family history of dementia, which is commonly associated with aging, and gut microbiota has not been described in studies, whereas patients with Alzheimer’s disease spectrum, including mild cognitive impairment, have reduced gut microbiota diversity and altered gut microbiota composition [69]. Regarding pets, numerous studies have already observed that early-life exposure to household pets [70] and current pet ownership are associated with changes in the human gut microbiota [71,72,73]. C2 had the most flexible work schedule, and only the minority of them were sleeping more on weekends, whereas in C3, the vast majority of subjects were working one shift, which is the most common work schedule in our society. A few studies highlighted the importance of circadian clocks for gut microbiota composition and function [74], and observed that night work alters gut microbiota composition [75]. For smoking, a systematic review observed a reduction in bacterial species diversity in smokers. Interestingly, the abundance of *Prevotella* was significantly increased in smokers, and the same was observed in the phylum Proteobacteria [76], while the abundance of *Faecalibacterium* was significantly lower in smokers [77]. Similarly, in our study, we observed that smoking was the most prevalent in C2, which had the highest abundance of *Prevotella 9*, and least present in C3, which had the highest abundance of *Faecalibacterium*. Regarding the use of antibiotics, which has been identified as a significant predictor, none of the subjects in C2 used antibiotics 3 to 5 months prior to their participation in the study. It has been clear for a long time that antibiotics induce changes in the composition and diversity of gut microbiota; however, after stopping their use, the gut microbiota returns to baseline within a few weeks [78].

The intensity of GI symptoms and the regularity of bowel movements were also identified as significant gut microbiota composition predictors. It has been observed that gut microbiota dysbiosis may contribute to irregular bowel movement and functional constipation [79]. The gut bacteria ferment nondigestible carbohydrates, produce flatulence, and can aggravate some GI symptoms [80]. Additionally, patients with flatulence and borborygmi have a poor tolerance of intestinal gas, which has been associated with gut microbiota instability [81].

Among psychological factors, significant gut microbiota composition predictors were subjective general health and mood, and symptoms of depression. Similar to our study, one of the most important factors that have been associated with gut microbiota is subjective mood, even in adults without mood disorders [82]. Numerous studies have observed an association between depression and gut microbiota composition, such as a higher abundance of proinflammatory species and a lower abundance of bacteria that produce SCFA [83,84,85]. In the present study, symptoms of depression were the least prevalent in C3, which has been characterized by a high abundance of *Faecalibacterium* that has been reported to improve depressive behavior. Lower abundances of *Faecalibacterium* have been observed in patients with depression [86], and its abundance has been positively associated with quality of life [87].

Significant gut microbiota composition predictors from the category of specific nutrients intake were the intake of SFA, sugars, free sugars, magnesium, iodine, and manganese. In mice, it has been observed that manganese is vital for proper maintenance of the intestinal barrier [88], but human studies are lacking. Most studies about the relationship between magnesium and gut microbiota have also been performed on animals; however, one study observed that magnesium supplements can modulate gut microbiota composition and the gut–brain axis in adults with GI functional disorders [89]. Subjects in C2, which was the group that most differed from all others, had the lowest intake of SFA and iodine. Similarly, it has been suggested that gut microbiota may play a role in the absorption of iodine, and the intake of iodine could have an important impact on gut microbiota [90]. Subjects in C4 had the highest intake of SFA and free sugars and the lowest intake of manganese. A systematic review observed that a high intake of SFA may negatively affect gut microbiota richness and diversity [91], whereas a high sugar intake can disrupt gut microbiota stability with a higher abundance of Proteobacteria, increased proinflammatory properties, and a decreased capacity to regulate epithelial integrity [92].

Overall, our findings suggest that lifestyle factors in combination with the intake of specific nutrients are more important predictors than just dietary pattern alone. Based on our model, 26 variables were crucial to very accurately (in 91%) predict in which cluster an individual’s microbiota was classified. Subjects’ microbiota composition can be classified in specific clusters not only depending on their nutrient intake, but also depending on their anthropometric measurements, the environment in which they live, living with pets, work schedule, family history, and psychological and other lifestyle factors. These factors can be causally, consequentially, or bidirectionally linked to gut microbiota composition. Some of the factors can be modified with changes in lifestyle, such as changes in the intake of specific nutrients or anthropometric measurements, while some, such as family history of dementia or the living environment, are nonmodifiable factors. This should be taken into account when developing strategies aiming to modulate gut microbiota composition.

## 5. Conclusions

Our aim was to investigate the relationship between four distinct dietary patterns (O, VE, V, and LCHF diet) and gut microbiota composition, and to evaluate if gut microbiota composition could be a useful indicator of a long-term dietary pattern. We observed many differences between the groups. At the phylum level, LCHF had the lowest abundance of Actinobacteria and highest abundance of Desulfobacterota. O had the highest abundance of Verrucomicrobiota, whereas V had the lowest. At the genera level, the predominant in all diet groups were *Bacteroides*, *Faecalibacterium*, *Prevotella 9*, and *Alistipes*. *Prevotella 9*, *Bifidobacterium*, *Haemophilus*, *Lachnospiraceae UCG-004*, *Subdoligranulum*, and *Anaerostipes* were predominant in V; *Ruminococcaceae CAG-352*, *Lachnospiraceae UCG-001*, and *Oscillospiraceae UCG-003* were predominant in VE; *Agathobacter*, *Lachnospiraceae ND3007*, *Victivallis*, *Ruminococcus*, *Rhodospirillales uncultured*, *Blautia*, and *Izemoplasmatales* were predominant in O; and *Alistipes*, *Ruminococcus torques*, *Lachnospiraceae uncultured*, *Odoribacter*, *Butyricimonas*, *Ruminococcaceae uncultured*, *Fusicatenibacter*, *Desulfovibrio*, and *Anaerosporobacter* were predominant in LCHF. However, after hierarchical clustering, we concluded that gut microbiota composition at the genus level is not a useful indicator to determine a subject’s dietary pattern, with the exception of a V diet that is represented by a high relative abundance of the genus *Prevotella 9*. Nevertheless, due to high interindividual variability, an individual could still be adhering to a V diet and not have this specific gut microbiota composition. The most important gut microbiota composition predictors were from the categories anthropometric measurements, serum biomarkers, lifestyle factors, GI symptoms, psychological factors, and specific nutrients intake. Thus, we can conclude that a combination of different lifestyle factors is more important to determine subjects’ gut microbiota composition than their dietary intake alone. With other lifestyle factors taken into account, we can predict subjects’ classification in specific clusters with 91% accuracy. There is no such thing as an “ideal” gut microbiota composition for human health; however, some gut bacterial genera are more related to different health markers. Our findings, which should be confirmed in a larger sample size of subjects, could serve to develop strategies to educate individuals about changes in lifestyle and specific nutrients intake, independent of their dietary pattern, with the aim to change some of the modifiable factors to classify into C3, instead of C1 or C4, which has been associated with favorable lipid and inflammatory profile.

## Figures and Tables

**Table 1:** Study subjects (*n* = 89).

	O (*n* = 24)	V (*n* = 24)	VE (*n* = 21)	LCHF (*n* = 20)	
**Gender**	**%**	**%**	**%**	**%**	***p*-value**
Males/Females	33.3/66.7	33.3/66.7	33.3/66.7	30.0/70.0	0.994
**Age**	**M (SD)**	**M (SD)**	**M (SD)**	**M (SD)**	***p*-value**
Age (years)	36.2 (10.4)	33.6 (9.6)	37.1 (10.8)	39.4 (6.9)	0.097
**Anthropometric measurements**	**M (SD)**	**M (SD)**	**M (SD)**	**M (SD)**	***p*-value**
BMI (kg/m^2^)	22.2 (3.0)	21.7 (2.2)	22.3 (2.4)	23.3 (3.2)	0.381
Waist circumference (cm)	75.5 (9.8)	74.5 (7.6)	76.2 (8.8)	76.8 (8.3)	0.845
Hip circumference (cm)	95.5 (5.5)	94.5 (5.1)	96.2 (5.8)	97.2 (7.1)	0.477
Fat mass (%)	21.8 (7.3)	19.7 (8.2)	22.0 (7.2)	21.5 (7.1)	0.713
Total body water (%)	57.6 (6.0)	58.7 (7.2)	57.2 (6.1)	57.9 (6.7)	0.890
Phase angle (°)	6.6 (1.0)	6.4 (0.9)	6.2 (0.8)	6.5 (1.0)	0.559
**Blood pressure**	**M (SD)**	**M (SD)**	**M (SD)**	**M (SD)**	***p*-value**
Systolic BP (mmHg)	119.5 (13.6)	124.2 (16.5)	124.6 (18.3)	121.8 (9.1)	0.619
Diastolic BP (mmHg)	76.4 (8.8)	77.1 (10.9)	77.6 (11.8)	77.4 (7.9)	0.890
**Family status**	**%**	**%**	**%**	**%**	***p*-value**
Single	29.2	29.2	19.0	10.0	0.367
In a relationship or married	70.8	70.8	81.0	90.0	
**Education**	**%**	**%**	**%**	**%**	***p*-value**
High school	25.0	29.2	28.6	35.0	0.708
Bachelor’s degree	50.0	62.5	61.9	50.0	
Master’s degree or PhD	25.0	8.3	9.5	15.0	
**Socioeconomic status**	**%**	**%**	**%**	**%**	***p*-value**
Employed	79.2	66.7	76.2	80.0	0.818
Unemployed/housewife	8.3	12.5	9.5	15.0	
Student	12.5	20.8	14.3	5.0	
**Work schedule**	**%**	**%**	**%**	**%**	***p*-value**
Not working	4.2	12.5	19.0	10.0	0.225
One-shift	50.0	41.7	52.4	75.0	
Two-shifts	37.5	25.0	14.3	10.0	
Flexible	8.3	20.8	14.3	5.0	
**Living with**	**%**	**%**	**%**	**%**	***p*-value**
Alone	16.7	20.8	14.3	5.0	0.220
With partner and/or children	62.5	54.2	61.9	85.0	
With parents	20.8	20.8	9.5	10.0	
With friends/roommates	0.0	4.2	14.3	0.0	
**Alcohol intake**	**M (SD)**	**M (SD)**	**M (SD)**	**M (SD)**	***p*-value**
Alcohol (units/week)	2.1 (3.8)	1.0 (1.3)	1.3 (1.8)	2.0 (3.6)	0.925
**Physical activity**	**M (SD)**	**M (SD)**	**M (SD)**	**M (SD)**	***p*-value**
IPAQ (MET/day)	11.7 (9.3)	11.8 (11.4)	9.7 (8.7)	7.5 (6.3)	0.443
**Other lifestyle factors influencing gut microbiota**	**%**	**%**	**%**	**%**	***p*-value**
Smoking	25.0	12.5	4.8	20.0	0.270
Psychoactive substances use	8.3	16.7	9.5	15.0	0.788
Use of antibiotics in the last year	20.9	25.0	0.0	5.0	0.104
Allergies	33.3	25.0	14.3	15.0	0.367
Vaginal birth	95.8	91.7	85.7	90.0	0.696
Having been breastfed	83.3	91.7	90.5	80.0	0.236
Growing up in a rural environment	29.2	75.0	61.9	60.0	0.012 *
Growing up with pets	41.7	91.7	61.9	70.3	0.003 *
Currently living with pets	54.2	54.2	57.1	55.0	0.997

O—omnivorous; V—vegan; VE—vegetarian; LCHF—low-carbohydrate, high-fat; *—statistically significant difference (chi-squared test); IPAQ—International Physical Activity Questionnaire.

**Table 2:** Serum biomarkers (*n* = 89).

Serum Biomarkers	O (*n* = 24)	V (*n* = 24)	VE (*n* = 21)	LCHF (*n* = 20)	*p*-Value
	M (SD)	M (SD)	M (SD)	M (SD)	
Glucose (mmol/L)	4.81 (0.48)	4.63 (0.37)	4.64 (0.48)	4.63 (0.58)	0.519
Cholesterol (mmol/L)	4.45 (0.72)	4.00 (0.92)	4.48 (0.88)	7.57 (4.67)	<0.001 ^b,c,d^
HDL (mmol/L)	1.89 (0.45)	1.58 (0.44)	1.86 (0.38)	2.16 (0.51)	0.001 ^c^
LDL (mmol/L)	2.84 (0.64)	2.67 (0.82)	2.94 (0.86)	5.87 (4.87)	<0.001 ^b,c,d^
TAG (mmol/L)	0.91 (0.61)	0.88 (0.42)	0.86 (0.32)	0.92 (0.72)	0.730
Iron (μmol/L)	27.79 (9.35)	22.95 (10.00)	18.11 (8.31)	16.35 (6.38)	<0.001 ^a,b^
AST (U/L)	22.90 (10.22)	21.54 (7.94)	19.34 (6.44)	18.94 (5.11)	0.461
Albumines (g/L)	46.45 (4.63)	46.15 (3.44)	46.45 (3.52)	45.39 (3.42)	0.778
Bilirubin (μmol/L)	9.74 (5.68)	8.88 (6.58)	9.23 (4.17)	6.77 (4.53)	0.157
CRP (mg/L)	1.21 (2.01)	0.57 (0.66)	0.62 (0.80)	0.73 (0.64)	0.631
LBP (μg/mL)	4.31 (1.20)	3.76 (1.60)	3.57 (1.60)	3.67 (1.95)	0.608
IL-6 (pg/mL)	2.22 (2.80)	1.45 (1.41)	1.21 (0.59)	2.47 (3.98)	0.935
TNF-α (pg/mL)	0.45 (0.27)	1.14 (2.99)	0.78 (0.64)	1.21 (1.93)	0.149

O—omnivorous; V—vegan; VE—vegetarian; LCHF—low-carbohydrate, high-fat; ^a^—O–VE; ^b^—O–LCHF; ^c^—V–LCHF; ^d^—VE–LCHF (post hoc tests); HDL—high-density lipoprotein; LDL—low-density lipoprotein; TAG—triacylglycerol; AST—aspartate aminotransferase; CRP—C-reactive protein; LBP—lipopolysaccharide binding protein; IL-6—interleukin-6; TNF-α—tumor necrosis factor-α.

**Table 3:** Dietary intake of macronutrients (*n* = 89).

	O (*n* = 24)	V (*n* = 24)	VE (*n* = 21)	LCHF (*n* = 20)		
**Energy**	**M (SD)**	**M (SD)**	**M (SD)**	**M (SD)**	***p*-value**	
Energy intake (kcal)	2162 (800)	2141 (716)	2143 (664)	1981 (568)	0.895	
**Protein**	**M (SD)**	**M (SD)**	**M (SD)**	**M (SD)**	***p*-value**	**RDI**
Total protein (%)	16.0 (3.1)	12.3 (2.8)	13.1 (3.7)	22.5 (5.7)	<0.001 ^a,c,d,e^	10–15
Plant protein (%)	5.9 (2.1)	11.6 (2.9)	8.0 (2.3)	1.9 (1.6)	<0.001 ^a,c,d,e^	
**Carbohydrates**	**M (SD)**	**M (SD)**	**M (SD)**	**M (SD)**	***p*-value**	**RDI**
Total carbohydrates (%)	46.6 (7.1)	59.0 (11.2)	50.5 (10.4)	9.4 (6.0)	<0.001 ^a,c,d,e^	>50
Sugars (%)	16.3 (5.2)	17.4 (8.3)	17.7 (5.7)	5.1 (3.9)	<0.001 ^c,d,e^	
Free sugars (%)	7.2 (4.0)	4.1 (3.6)	6.4 (2.6)	1.6 (2.4)	<0.001 ^a,c,e^	<10
Dietary fiber (g)	27.6 (17.6)	55.4 (48.1)	35.1 (13.9)	16.3 (22.9)	<0.001 ^a,c,d,e^	>30
**Fats**	**M (SD)**	**M (SD)**	**M (SD)**	**M (SD)**	***p*-value**	**RDI**
Total fats (%)	35.5 (7.1)	27.6 (9.8)	36.1 (9.3)	66.2 (8.2)	<0.001 ^a,c,d,e,f^	25–30
SFA (%)	10.7 (2.7)	5.8 (3.2)	9.8 (3.5)	25.1 (6.1)	<0.001 ^a,c,d,e,f^	<10
MUFA (%)	10.2 (3.8)	9.6 (4.7)	10.9 (5.1)	22.0 (6.7)	<0.001 ^c,d,e^	>10
ω-3 PUFA (%)	0.6 (0.6)	0.7 (0.7)	0.7 (0.7)	1.1 (0.7)	0.008 ^c,d^	0.5
ω-6 PUFA (%)	2.9 (2.1)	3.1 (2.1)	3.9 (2.6)	4.6 (1.7)	0.010 ^c^	2.5
ω-3/ω-6 PUFA (ratio)	0.2 (0.2)	0.4 (0.7)	0.2 (0.2)	0.3 (0.2)	0.333	>0.2
EPA (mg)	131.7 (218.4)	46.6 (152.7)	72.5 (154.6)	370.3 (533.5)	<0.001 ^a,b,d,e^	
DHA (mg)	268.9 (449.4)	47.7 (129.3)	104.8 (223.5)	530.2 (675.9)	<0.001 ^a,b,d,e^	
Cholesterol (mg)	322.6 (181.8)	12.4 (18.2)	137.9 (126.1)	1106.0 (529.9)	<0.001 ^a,c,d,e,f^	
**Use of probiotics**	**%**	**%**	**%**	**%**	***p*-value**	
Probiotics	16.7	29.2	4.8	0.0	0.021 *	
**Adherence to Mediterranean diet**	**M (SD)**	**M (SD)**	**M (SD)**	**M (SD)**	***p*-value**	
MEDAS (score)	6.8 (2.3)	8.8 (1.7)	8.2 (1.8)	5.9 (1.8)	<0.001 ^a,d,e^	

O—omnivorous; V—vegan; VE—vegetarian; LCHF—low-carbohydrate; high-fat; ^a^—O–V; ^b^—O–VE; ^c^—O–LCHF; ^d^—V–LCHF; ^e^—VE–LCHF; ^f^—V–VE (post hoc tests); *—statistically significant difference (chi-squared test); FA—fatty acids; SFA—saturated fatty acids; MUFA—monounsaturated fatty acids; PUFA—polyunsaturated fatty acids; MEDAS—Mediterranean Diet Adherence Score; RDI—recommended dietary intake (for Slovenian population).

**Table 4:** GI symptoms and stool consistency (*n* = 89).

	O (*n* = 24)	V (*n* = 24)	VE (*n* = 21)	LCHF (*n* = 20)	
**Stool consistency**	**M (SD)**	**M (SD)**	**M (SD)**	**M (SD)**	***p*-value**
Bristol stool scale (score)	3.9 (0.9)	4.2 (1.2)	3.8 (0.7)	3.3 (1.0)	0.019 ^b^
**GI symptoms**	**M (SD)**	**M (SD)**	**M (SD)**	**M (SD)**	***p*-value**
Nausea (frequency)	0.5 (0.8)	0.4 (0.7)	0.3 (0.7)	0.2 (0.4)	0.415
Bloating (frequency)	1.6 (1.1)	1.7 (1.0)	0.9 (0.9)	1.0 (1.1)	0.018 *
Borborygmi (frequency)	1.3 (1.0)	1.5 (1.1)	1.1 (0.9)	0.9 (1.0)	0.186
Abdominal pain (frequency)	0.6 (0.7)	0.8 (0.8)	0.5 (0.8)	0.4 (0.7)	0.151
Flatulence (frequency)	1.7 (0.9)	1.9 (0.9)	1.5 (0.9)	0.8 (0.9)	0.002 ^a,b^
Heartburn (frequency)	0.5 (0.9)	0.7 (1.0)	0.6 (1.0)	0.4 (0.6)	0.763

O—omnivorous; V—vegan; VE—vegetarian; LCHF—low-carbohydrate, high-fat; ^a^—O–LCHF; ^b^—V–LCHF (post hoc tests); *—statistically significant difference (ANOVA).

**Table 5:** Gut microbial phyla (*n* = 89).

	**O (*n* = 24)**	**V (*n* = 24)**	**VE (*n* = 21)**	**LCHF (*n* = 20)**	
**Phylum**	**M (SD)**	**M (SD)**	**M (SD)**	**M (SD)**	***p*-value**
Firmicutes (%)	45.41 (16.34)	42.07 (16.33)	47.13 (17.24)	42.56 (15.19)	0.702
Bacteroidota (%)	47.37 (17.64)	51.67 (18.19)	46.35 (16.61)	51.74 (14.88)	0.611
Proteobacteria (%)	4.19 (2.15)	4.06 (3.18)	4.34 (3.79)	3.61 (2.58)	0.691
Verrucomicrobiota (%)	1.02 (1.12)	0.36 (0.76)	0.69 (0.89)	0.51 (0.65)	0.020 ^a^
Cyanobacteria (%)	1.00 (3.00)	0.45 (0.84)	0.91 (1.60)	0.84 (1.69)	0.577
Actinobacteria (%)	0.79 (1.11)	0.96 (1.64)	0.27 (0.28)	0.05 (0.06)	<0.001 ^b,c,d^
Desulfobacterota (%)	0.21 (0.23)	0.37 (0.67)	0.32 (0.54)	0.66 (0.67)	0.018 ^c,d^
**Gut microbiota α-diversity**	**M (SD)**	**M (SD)**	**M (SD)**	**M (SD)**	***p*-value**
Shannon index	3.27 (0.48)	2.85 (0.73)	3.27 (0.49)	3.33 (0.46)	0.090

O—omnivorous; V—vegan; VE—vegetarian; LCHF—low-carbohydrate, high-fat; ^a^—O–V; ^b^—O–LCHF; ^c^—V–LCHF; ^d^—VE–LCHF (post hoc tests).

**Table 6:** Significant gut microbiota composition cluster predictors (*n* = 89).

Predictor	C1 (*n* = 28)	C2 (*n* = 8)	C3 (*n* = 33)	C4 (*n* = 20)	
**Anthropometric measurements**	**M (SD)**	**M (SD)**	**M (SD)**	**M (SD)**	***p-*** **value**
Hip circumference (cm)	98.3 (5.9)	94.3 (4.9)	95.7 (5.6)	92.8 (5.1)	0.008 ^b^
Phase angle (°)	6.6 (0.9)	7.2 (0.7)	6.3 (0.8)	6.3 (0.9)	0.033 ^c^
Diastolic blood pressure (mm Hg)	77.1 (8.9)	73.9 (5.8)	78.8 (11.3)	75.6 (9.8)	0.747
**Serum biomarkers**	**M (SD)**	**M (SD)**	**M (SD)**	**M (SD)**	***p-*** **value**
Serum TAG (mmol/L)	1.10 (0.80)	0.79 (0.24)	0.72 (0.26)	0.93 (0.36)	0.092
Serum LBP (μg/mL)	4.24 (1.61)	3.97 (1.46)	3.58 (1.76)	3.68 (1.29)	0.500
**Subject’s lifestyle factors**	**%**	**%**	**%**	**%**	***p-*** **value**
Growing up with pets	78.6	100.0	48.5	65.0	0.014 *
Currently having pets	53.6	37.5	60.6	55.0	0.698
Regular bowel movement	89.3	87.5	69.7	75.0	0.262
Smoking	17.9	25.0	9.1	20.0	0.575
Family history of dementia	14.3	37.5	9.1	35.1	0.055
Sleeping more on weekends	57.1	37.5	63.6	55.0	0.600
Work schedule (not working/one shift/two shifts/flexible)	10.7/50.0/32.1/7.1	25.0/12.5/15.5/50.0	3.0/69.7/18.2/9.1	20.0/50.0/20.0/10.0	0.013 *
Last use of antibiotics (>1 year ago or never/6–12 months ago/3–5 months ago)	92.9/0.0/7.1	62.5/37.5/0.0	87.9/9.1/3.0	85.0/10.0/5.0	0.076
Parents alive (both/one/no one)	78.6/7.1/14.3	100.0/0.0/0.0	72.7/27.3/0.0	90.0/5.0/5.0	0.024 *
**GI symptoms**	**M (SD)**	**M (SD)**	**M (SD)**	**M (SD)**	***p-*** **value**
Borborygmi (intensity)	0.6 (0.7)	0.9 (0.6)	0.9 (0.8)	1.0 (0.8)	0.281
Flatulence (intensity)	1.2 (0.8)	1.0 (0.8)	1.2 (0.8)	1.4 (0.8)	0.694
Heartburn (intensity)	0.3 (0.5)	0.6 (1.1)	0.5 (0.7)	0.6 (0.8)	0.503
**Psychological factors**	**M (SD)**	**M (SD)**	**M (SD)**	**M (SD)**	***p-*** **value**
Subjective general health (score)	4.6 (0.6)	4.1 (0.4)	4.3 (0.6)	4.4 (0.7)	0.172
Subjective mood (score)	4.2 (0.9)	3.8 (0.7)	4.0 (0.9)	3.7 (0.8)	0.062
Symptoms of depression (score)	8.5 (9.4)	9.9 (8.0)	7.7 (5.3)	10.3 (5.6)	0.206
**Specific nutrients intake**	**M (SD)**	**M (SD)**	**M (SD)**	**M (SD)**	***p-*** **value**
SFA (%)	13.5 (9.0)	5.2 (2.3)	12.1 (6.5)	14.2 (9.5)	0.008 ^a,c,d^
Sugars (%)	14.1 (9.6)	12.6 (4.1)	14.1 (6.7)	16.2 (8.2)	0.421
Free sugars (%)	4.3 (3.6)	3.4 (2.1)	4.9 (3.5)	6.3 (5.1)	0.429
Magnesium (mg)	708.8 (673.3)	695.6 (425.2)	515.4 (479.2)	415.7 (217.6)	0.101
Iodine (µg)	78.5 (42.9)	43.4 (27.2)	113.3 (68.9)	96.2 (47.5)	0.006 ^c,d^
Manganese (mg)	14.5 (35.5)	9.4 (6.6)	8.3 (12.3)	5.1 (3.4)	0.144

^a^—C1–C2; ^b^—C1–C4; ^c^—C2–C3; ^d^—C2–C4 (post hoc tests); *—statistically significant difference (chi-squared test); TAG—triacylglycerol; LBP—lipopolysaccharide binding protein; SFA—saturated fatty acids.

## Data Availability

The authors confirm that the data supporting the findings of this study are available within the article and the raw sequencing results can be accessed with the accession number PRJNA944627 (http://www.ncbi.nlm.nih.gov/bioproject/944627). The additional data are available on request from the corresponding author.
